# Experimental Evidence for Reduced Rodent Diversity Causing Increased Hantavirus Prevalence

**DOI:** 10.1371/journal.pone.0005461

**Published:** 2009-05-06

**Authors:** Gerardo Suzán, Erika Marcé, J. Tomasz Giermakowski, James N. Mills, Gerardo Ceballos, Richard S. Ostfeld, Blas Armién, Juan M. Pascale, Terry L. Yates

**Affiliations:** 1 Museum of Southwestern Biology and Department of Biology, University of New Mexico, Albuquerque, New Mexico, United States of America; 2 Division of Viral and Rickettsial Diseases, Centers for Disease Control and Prevention, Atlanta, Georgia, United States of America; 3 Instituto de Ecología, Universidad Nacional Autónoma de México, Ciudad Universitaria, México, Distrito Federal, México; 4 Cary Institute of Ecosystem Studies, Millbrook, New York, United States of America; 5 Instituto Conmemorativo GORGAS, Ciudad de Panamá, Panamá; University of Lancaster, United Kingdom

## Abstract

Emerging and re-emerging infectious diseases have become a major global environmental problem with important public health, economic, and political consequences. The etiologic agents of most emerging infectious diseases are zoonotic, and anthropogenic environmental changes that affect wildlife communities are increasingly implicated in disease emergence and spread. Although increased disease incidence has been correlated with biodiversity loss for several zoonoses, experimental tests in these systems are lacking. We manipulated small-mammal biodiversity by removing non-reservoir species in replicated field plots in Panama, where zoonotic hantaviruses are endemic. Both infection prevalence of hantaviruses in wild reservoir (rodent) populations and reservoir population density increased where small-mammal species diversity was reduced. Regardless of other variables that affect the prevalence of directly transmitted infections in natural communities, high biodiversity is important in reducing transmission of zoonotic pathogens among wildlife hosts. Our results have wide applications in both conservation biology and infectious disease management.

## Introduction

The loss of biological diversity is an accelerating process with profound consequences for the ability of ecosystems to provide services to human societies. Ecosystem provision of services such as food production, water filtration, and protection against floods and droughts is reduced when biological diversity is lost [1]. High biological diversity also is hypothesized to protect against human and wildlife diseases [2], [3], and reduced disease spread or prevalence has been recognized as an ecosystem service [4]. Many zoonoses and wildlife diseases, such as Lyme disease [5], West Nile Virus [6] and SARS [7], are caused by generalist pathogens (i.e. those that readily infect >1 host species) [8], for which relatively few, common species act as reservoirs [5]. In such disease systems, high species diversity within the host community is predicted to deflect pathogen transmission away from the primary reservoirs and toward hosts that act as a sink for the pathogen [5], [9], or to suppress the abundance of the primary reservoirs, which will reduce density-dependent transmission rates [10]. In both cases, high diversity is correlated with low disease risk or prevalence. With the exception of fungal diseases of plants [11]–[13] experimental assessments of the role of species diversity in disease dynamics are lacking.

Hantaviruses, which are transmitted to humans by inhalation of the aerosolized virus or by direct contact, are hosted by a large variety of rodent species of the family Muridae in Europe, Asia and the Americas [14]. They cause hemorrhagic fever with renal syndrome in Asia and Europe and hantavirus pulmonary syndrome (HPS) in the Americas. Although HPS incidence in the United States is low, case fatality is high (35% [15]). Tropical regions in the Americas provide an ideal place to test the effect of diversity on infection dynamics because of high rodent species diversity, high deforestation and habitat loss rates affecting that diversity, and high prevalence of emerging and re-emerging diseases such as HPS. In Central America, two rodent species, *Oligoryzomys fulvescens* and *Zygodontomys brevicauda*, have been reported as competent reservoirs (i.e. a species that maintains and readily transmits the virus to other animals including humans) for Choclo and Calabazo viruses [16]. These two species become the dominant rodents in deforested areas, where small mammal diversity is reduced, usually occupying habitats close to humans [17]. Some evidence suggests that forest-field edges are particularly risky locations for human exposure to zoonotic pathogens [18], [19].

To investigate the effect of species diversity on hantavirus infection we carried out an experimental study of the small mammal community in the Azuero Peninsula in Panama, manipulating the species richness and population densities of host and non-host species in experimental plots at edges of small forest fragments. We divided small mammal species into *competent reservoir species*, and *non-reservoir species*, which may become incidentally infected, but are not known to maintain or transmit hantaviruses. We sought to mimic natural patterns of species diversity loss by experimentally removing non-reservoir species. We hypothesized that experimentally reduced small-mammal diversity would cause hantavirus infection prevalence in the competent reservoir species to increase relative to unmanipulated control communities. We further hypothesized that such an increase in infection prevalence would be caused by two independent processes: (i) an increase in average population density of the competent reservoir species; and (ii) an increase in intraspecific transmission irrespective of population density of the competent reservoir species. These two mechanisms correspond, respectively, to the “susceptible host regulation” and “encounter reduction” modes by which high diversity reduces pathogen transmission [2]. In the first mode, if reservoir populations are regulated by a diverse assemblage of potential competitors, density-dependent pathogen transmission should be reduced. In the second, if a diverse assemblage of species reduces the frequency of intraspecific interactions that can elicit transmission (e.g., by changing reservoir behavior), frequency-dependent pathogen transmission should be reduced.

Comparative (non-experimental) tests of these hypotheses are typically unable to directly assign cause-effect relationships and usually cannot implicate mechanisms underlying correlations between diversity and disease dynamics. Therefore, to evaluate the effects of small mammal diversity on hantavirus infection dynamics, we implemented a mark-recapture experiment with selective species removal at replicated field sites.

## Methods

### Ethics statement

Research was approved by the University of New Mexico Institutional Animal Care and Use Committee.

### Study area and trapping

The study area is located in the southern part of Azuero Peninsula, in southwestern Panama (−80°59′W, 7°67′N). Azuero Peninsula is mostly a transformed agricultural landscape with remnants of tropical dry forest and low human densities. The native vegetation consists predominantly of deciduous trees with some perennial and succulent species, with mangrove swamp near the coast; grasslands consist of several introduced pasture grasses. While most of the country has a hot and humid tropical climate with cooler temperatures in higher elevations, mean annual rainfall is least along the central Pacific coast, where rainfall is approximately 1200 mm yr^−1^. On the Azuero Peninsula, the climate is extremely arid during the dry season (January to April), and several weeks can go by with no rain at all. Topographically, elevation in the study area varies from sea level to over 1500 m, with highest elevations and mountainous terrain concentrated along the western side of the peninsula, where the terrain is generally steep and broken, with slope gradients commonly >45°.

**Figure 1:**
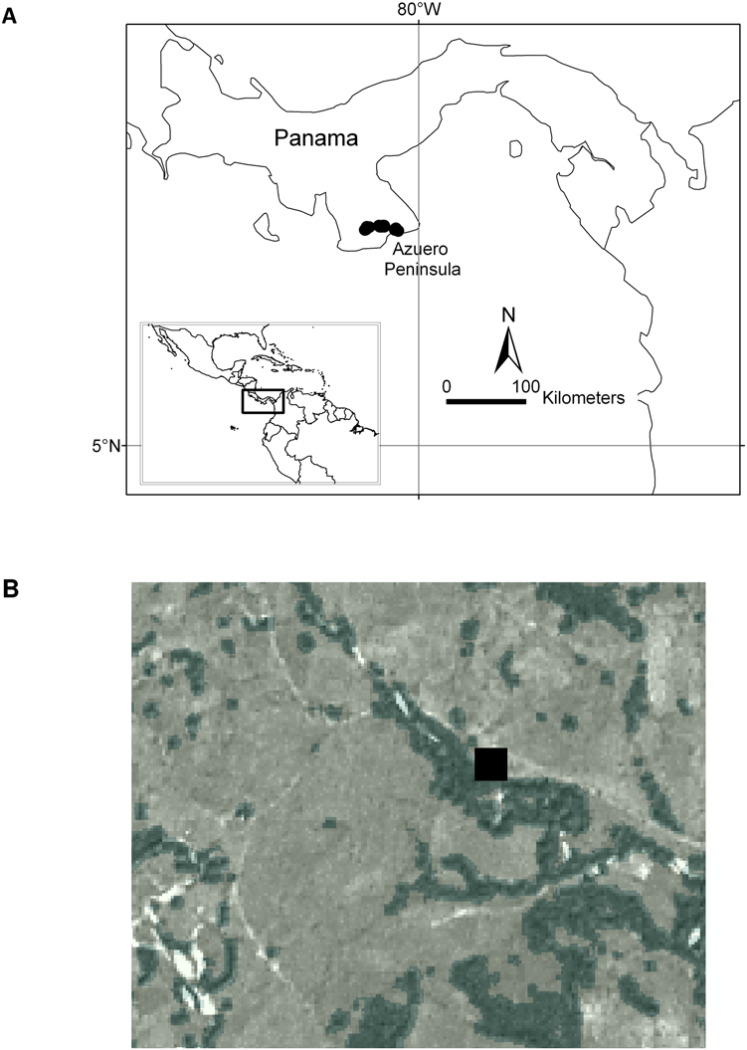
Study sites in the Azuero Peninsula Panama. The 24 study sites were located in edges of small fragments of forest in the Azuero Peninsula Panama. The satellite image indicates, as an example, the location of El Cortezo site; note the fragmented nature of the landscape and the location of the sampling grid (black square) in the forest – grassland edge.

We selected 24 sites in four general localities that shared similar environmental conditions and were distributed along an east-west axis in the southern part of the peninsula (Figure 1A). Distances between nearest neighbour sites ranged from 175 m to 2987 m (mean = 539 m, SD = 655 m). All selected sites were forest edges that we defined as an abrupt transition between two relatively homogeneous ecosystems very common in human-dominated tropical areas: pastures and primary or secondary tropical forests (Figure 1B).

### Landscape characteristics

To measure and control for landscape similarity between all sites, we quantified landscape fragmentation characteristics, such as edge density and number of isolated fragments. These landscape metrics were produced from a layer derived from a high resolution (10 m) panchromatic SPOT image (Satellite Pour l'Observation de la Terre, designed and operated by the Centre National d'Etudes Spatiales, France). The image was obtained from the U.S. National Geospatial-Intelligence Agency (formerly the National Imagery and Mapping Agency). We classified the panchromatic 10 m SPOT image into forested and non-forested areas using supervised classification in Erdas Imagine ver. 9.1 (Erdas Inc., Atlanta, GA, USA). Although we did not perform a quantitative assessment of the accuracy of the classification, a qualitative examination of predicted forest fragments matched very well with the senior author's field notes. Once the image was classified into forested patches, we used the Patch Analyst ArcView extension [20] to calculate landscape characteristics for all trapping sites at a scale of 222 m. This scale was based on the maximal recapture distance of an individual captured in this study. For each trapping site, we obtained 13 variables describing landscape configuration characteristics that were then compared between experimental and control sites.

### Trapping grids

For each of four localities, we set up two control and four experimental sites for a total of 24 sites. At each site we set a trapping grid consisting of a 7×7 square array of Sherman traps (8×8×23 cm; H. B. Sherman Traps, Inc., Tallahassee, Florida, USA) at 10 m intervals. Each trapping session lasted three nights, with a revisit time to the same site of about one month from June 27 to November 20, 2003. The central trap line in each grid was centred on the edge of the forest and the traps were baited with peanut butter and seeds. At control sites all individuals captured were measured, ear-tagged and released at capture site, and in addition, whole blood was taken from the retrorbital sinus of the eye using a heparinized capillary tube and Nobuto strips. Blood was tested by a strip immunoblot assay for detection of antibodies to SNV nucleocapsid (N) antigen [21], [22]. At experimental sites, all non-reservoir species were removed during live-trapping sessions, leaving only *Zygodontomys brevicauda* and *Oligoryzomys fulvescens* which also were ear-tagged, bled, and measured.

### Species diversity and evenness

The Simpson index of diversity, which is a measure that accounts for both richness and proportion of each species, was used to estimate diversity at each site. Simpson Index is considered a dominance index because it weights towards the abundance of the most common species but also gives the probability that any two individuals drawn at random from an infinitely large community belong to different species. The bias-corrected form of Simpson Index is:
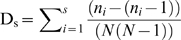
where *n_i_* is the number of individuals in the ith species. Since D_s_ and diversity are negatively related, Simpson's index is usually expressed as either reciprocal or complementary forms (1/D or 1 -D), we used the reciprocal 1/D Simpson index.

### Unbalanced design

We established an unbalanced designed (more experimental than control sites) to increase the probability of capturing hantavirus reservoir species and our power to detect an effect of species removal on hantavirus dynamics. This is based on previous sampling surveys at National Parks in Panama in which hantavirus reservoirs were relatively scarce. For all analyses comparing control and experimental sites we used means of values from all 16 experimental and eight experimental sites. To evaluate the effect of the unbalanced design, we also performed the same analyses using only eight randomly selected experimental sites (from the set of 16), but the results were consistent with those using all sites. In addition, different random sets of eight experimental sites produced the same final results, thus the unbalanced design did not affect the comparisons.

## Results

Control and experimental (species removal) sites did not differ in any of the measured landscape characteristics describing forested land cover surrounding the sites. All sites had similar patch sizes of forest, as well as length of patch edge and patch shape (two-sample t-tests, P>0.20). Overall, all sites were at similar elevations (mean = 87 m, SD = 36 m) although the topographic slope surrounding the sites (expressed as a percentage) ranged from 0 to 39%. However, no significant differences occurred between control or experimental sites for either elevation or slope (two-sample t-tests, P>0.7). Small mammal species diversity was relatively high in the study region and within most sites. The nine species (three families) captured were *Zygodontomys brevicauda*, *Liomys adspersus*, *Sigmodon hispidus*, *Oryzomys talamancae*, *Proechimys semispinosus*, *Oligoryzomys fulvescens*, *Mus musculus*, *Reithrodontomys darienensis*, and *Didelphis marsupialis*. We captured 905 individuals, 1460 times, which included 555 recaptures. The overall hantavirus antibody prevalence was high (124/905 = 13.7%). Five of 9 species (55%) had individuals with detectable antibody to a hantavirus. Most positive individuals (99 of the 124 positives, or 80%) were *Z. brevicauda*, although the highest prevalence was observed in *O. fulvescens* (33% of 3). Control and experimental sites had similar species diversity (Simpson diversity index) at the beginning of the study (ANOVA F = 0.470, Df = 1, P>0.5).

**Figure 2:**
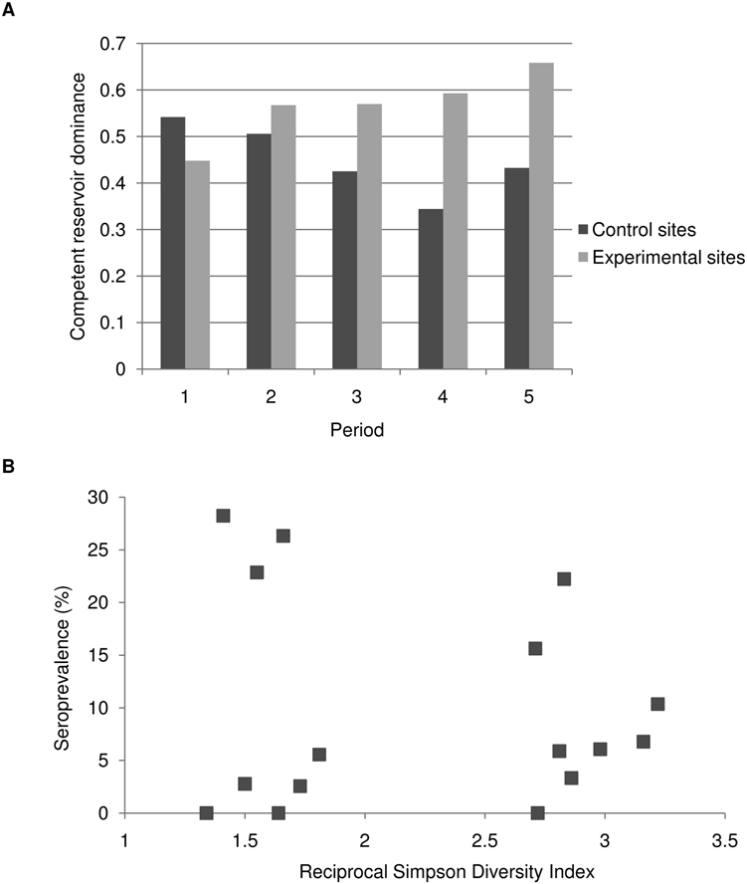
A. Hantavirus reservoir dominance, defined as the proportion of the community consisting of competent reservoir species, in control (unmanipulated) and experimental (non-reservoir species removed) sites during the sequential trapping periods. B. Relationship between mean hantavirus seroprevalence and species diversity (expressed as reciprocal Simpson's Index) within experimental plots, where diversity was manipulated.

At the beginning of the study, population densities of competent reservoir species were similar between control and experimental sites. Removal of non-reservoir species was associated with increases in both abundance and seroprevalence in competent reservoir populations. The population densities of *Z. brevicauda* remained relatively stable at the experimental sites. In contrast, there was a steady, significant decrease in population densities at the control sites (R = 0.397, P<0.01). Although the relative abundance of *Z. brevicauda* was initially slightly lower (50% of individuals in the community) in the treatment when compared with the control (54%) sites, the relative abundance (dominance) of this species increased steadily throughout the study period, and at the end it was significantly higher in the experimental sites (65 vs 48%, Z = 2.09, P = 0.036; Figure 2A). The prediction that population density of the competent reservoir species would be higher where diversity was reduced, was supported. These results suggest that populations of *Z. brevicauda* are regulated, at least in part, by the presence of other small mammals with which they probably compete. Higher overall and relative abundance of reservoir populations in experimental compared to control plots support the “susceptible host regulation” [2] mechanism by which diversity can reduce disease transmission (Figure 2B).

**Figure 3:**
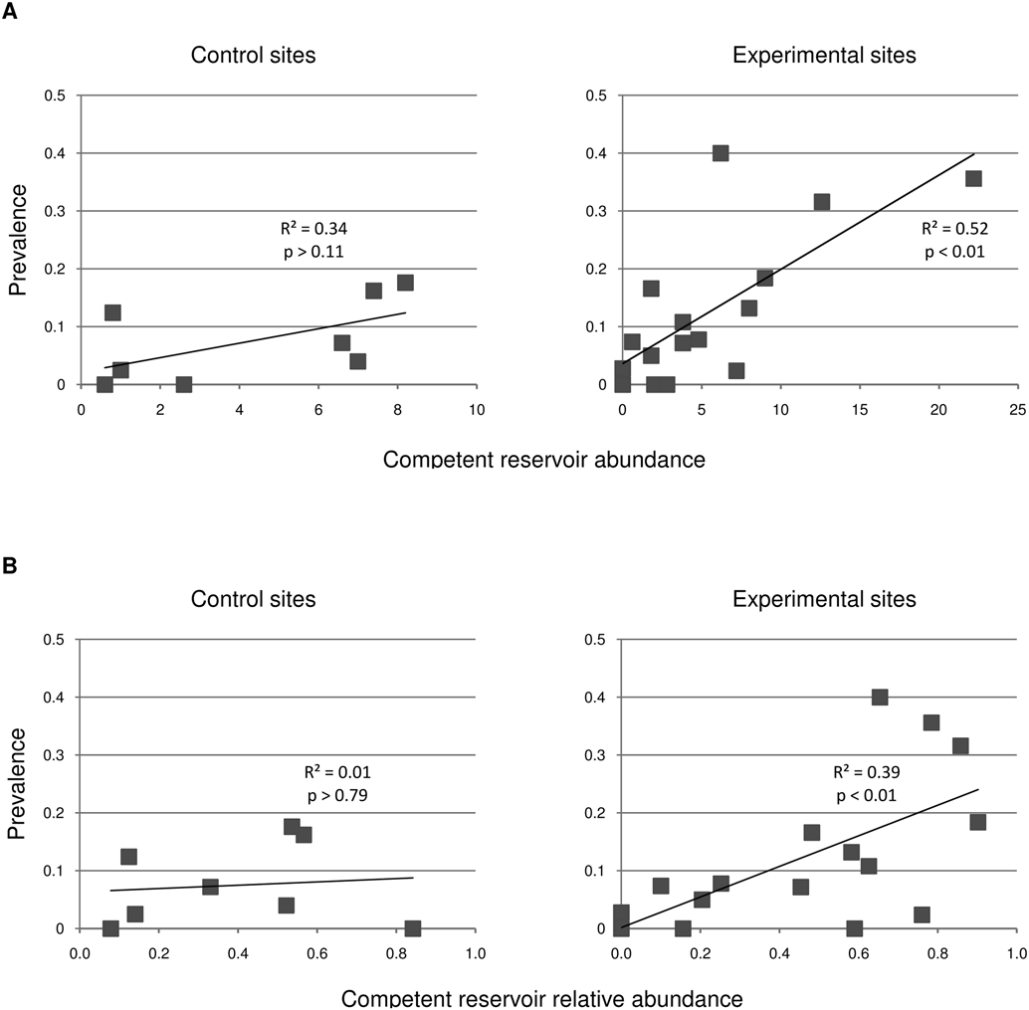
Relationship between competent reservoir abundances (*Z. brevicauda* and *O. fulvescens* and seroprevalence for hantaviruses control (unmanipulated) and experimental (non-reservoir species removed) sites. Data are averaged over all sampling periods per site and are those of absolute abundances (A and B) and relative abundances (C and D). Linear regression analyses revealed a statistically significant correlation between abundance and seroprevalence in experimental sites.

**Figure 4:**
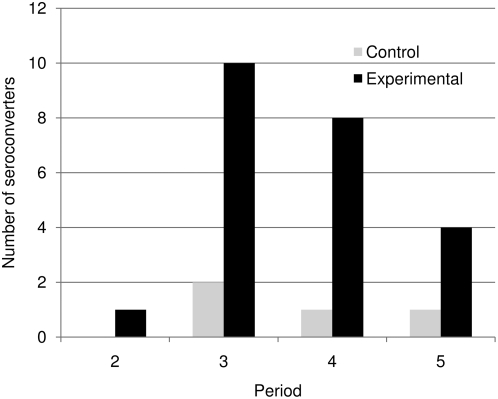
Number of individual *Z. brevicauda* and *O. fulvescens* seroconverting (transitioning from seropositive to seronegative) in experimental (non-reservoirs removed) and control (unmanipulated) plots.

We observed a strong positive correlation between both the relative abundance and total abundance of reservoir species in experimental sites and seroprevalence in those sites (Figures 3A and 3B). At the beginning of the study, the infection prevalence was 10% in experimental sites and 6% in control sites, but the differences were not statistically significant. By the end of the study, however, we observed 25% positive individuals in treatment sites and 14% in control sites (Table 1). This effect was stronger on those experimental sites that had an initially high species richness. Indeed, if the two experimental sites that had only one or two species at the beginning of the study are removed from the analysis, infection prevalence in treatment sites was 35%, more than twice as high as in the control sites (R = 0.23, P = 0.05). Experimental sites showed a stronger increase in seroprevalence over time than did control sites (Table 1). Log-linear analysis of a three-way contingency table (Table 1) showed a significant interactive effect of treatment (experimental and control) and period (before and after removals) on seroprevalence (G^2^ = 14.96, df = 4, P<0.005). In addition, rates of seroconversion were markedly higher in the experimental than control sites (Figure 4). Of the total seroconverters, i.e. individuals transitioning from negative to positive, 85% were found in the experimental sites. Practically all (96%) seroconverting individuals were *Z. brevicauda*.

**Table 1:** Number of seropositive and seronegative individual animals in control (unmanipulated) and experimental (non-reservoirs removed) sites before and after the removal treatment.

*Hantavirus negative*		*Hantavirus positive*	
Before	After	Before	After
90	32	6	5
189	59	21	20

## Discussion

As we predicted, there was a clear negative effect of high species diversity on both abundance of reservoir hosts and their infection prevalence with hantavirus. The higher seroprevalence and seroconversion rates in experimental (non-reservoirs removed) plots suggest that high small-mammal diversity reduces encounter rates between infected and susceptible hosts (the “encounter reduction” mode of the “dilution effect”[2]). These mechanisms potentially explaining the amplification of the infection in experimental sites are tied to fundamental principles in epidemiology. First, transmission rates and the success of pathogen reproduction are often proportional to the abundance of the host [23], [24]. To the extent that high diversity regulates abundance of the primary reservoir species, diversity should therefore reduce pathogen transmission and disease risk. Second, given that the prevalence of infection was reduced in high-diversity plots independent of the absolute density of reservoir hosts, our results suggest that decreased reservoir relative abundances, at higher species diversity, decreases pathogen transmission rate. Our experimental results also are consistent with epidemiological models in which adding a single non-reservoir species to an initial “community” consisting solely of the reservoir host reduces infection prevalence in the reservoir, sometimes to zero [25].

In the hantavirus system, as seems to be the case for other zoonotic diseases, such as Lyme disease, West Nile fever, leishmaniasis, and others, the primary reservoirs are ecological generalists that respond favorably to anthropogenic or natural forces that reduce species diversity. With respect to hantavirus outbreaks in central America, populations of *Z. brevicauda* appear to have been enhanced by habitat deterioration and the absence of predators (as a result of illegal hunting), while their tolerance to different and changing habitats allows them to dominate in degraded communities [18], [25], [26]. A somewhat different pattern, however, appears to exist for Sin Nombre hantavirus (SNV) in its reservoir host, *Peromyscus maniculatus*, in the North American west. A recent study [27] found a negative correlation between habitat disturbance (from off-road motor vehicles) and both abundance and seroprevalence for SNV in *P. maniculatus*. However, this study did not report on the effect of disturbance on small-mammal diversity.

Correlations between high host diversity and low rates of pathogen transmission or disease risk have been found for several other zoonotic diseases, including Lyme disease [28], [29], West Nile fever [6], [30], [31], and another hantavirus, Puumala virus [32]. Experimental evidence supporting a protective role of host diversity also has been gathered recently in diseases of wildlife [33] and freshwater plankton [34].

Results of our study provide the first experimental evidence, to our knowledge, for high biological diversity reducing pathogen transmission in a zoonotic disease, and point to the operation of two distinct mechanisms underlying this relationship. Strikingly similar results have been found in experimental studies in which fungal pathogen prevalence in herbaceous plants decreased with increasing plant species diversity [11], [13]. Experimental studies in other systems, combined with longer-term studies and models, will elucidate the degree to which this protective effect of high biological diversity is general. We suggest that the protection of human health should be added to the list of ecosystem services provided by high biodiversity.
